# Obtaining informed consent for clinical tumor and germline exome sequencing of newly diagnosed childhood cancer patients

**DOI:** 10.1186/s13073-014-0069-3

**Published:** 2014-09-17

**Authors:** Sarah Scollon, Katie Bergstrom, Robin A Kerstein, Tao Wang, Susan G Hilsenbeck, Uma Ramamurthy, Richard A Gibbs, Christine M Eng, Murali M Chintagumpala, Stacey L Berg, Laurence B McCullough, Amy L McGuire, Sharon E Plon, D Williams Parsons

**Affiliations:** Texas Children’s Cancer Center, 6701 Fannin Street #1400, Houston, TX 77030 USA; Department of Pediatrics, Baylor College of Medicine, One Baylor Plaza, Houston, TX 77030 USA; Dan L. Duncan Cancer Center, Baylor College of Medicine, One Baylor Plaza, Houston, TX 77030 USA; Dan L. Duncan Institute for Clinical and Translational Research, Baylor College of Medicine, One Baylor Plaza, Houston, TX 77030 USA; Human Genome Sequencing Center, Baylor College of Medicine, One Baylor Plaza, Houston, TX 77030 USA; Department of Molecular and Human Genetics, Baylor College of Medicine, One Baylor Plaza, Houston, TX 77030 USA; Center for Medical Ethics and Health Policy, Baylor College of Medicine, One Baylor Plaza, Houston, TX 77030 USA

## Abstract

**Background:**

Effectively educating families about the risks and benefits of genomic tests such as whole exome sequencing (WES) offers numerous challenges, including the complexity of test results and potential loss of privacy. Research on best practices for obtaining informed consent (IC) in a variety of clinical settings is needed. The BASIC3 study of clinical tumor and germline WES in an ethnically diverse cohort of newly diagnosed pediatric cancer patients offers the opportunity to study the IC process in the setting of critical illness. We report on our experience for the first 100 families enrolled, including study participation rates, reasons for declining enrollment, assessment of clinical and demographic factors that might impact study enrollment, and preferences of parents for participation in optional genomics study procedures.

**Methods:**

A specifically trained IC team offered study enrollment to parents of eligible children for procedures including clinical tumor and germline WES with results deposited in the medical record and disclosure of both diagnostic and incidental results to the family. Optional study procedures were also offered, such as receiving recessive carrier status and deposition of data into research databases. Stated reasons for declining participation were recorded. Clinical and demographic data were collected and comparisons made between enrolled and non-enrolled patients.

**Results:**

Over 15 months, 100 of 121 (83%) eligible families elected to enroll in the study. No significant differences in enrollment were detected based on factors such as race, ethnicity, use of Spanish interpreters and Spanish consent forms, and tumor features (central nervous system versus non-central nervous system, availability of tumor for WES). The most common reason provided for declining enrollment (10% of families) was being overwhelmed by the new cancer diagnosis. Risks specific to clinical genomics, such as privacy concerns, were less commonly reported (5.5%). More than 85% of parents consented to each of the optional study procedures.

**Conclusions:**

An IC process was developed that utilizes a specialized IC team, active communication with the oncology team, and an emphasis on scheduling flexibility. Most parents were willing to participate in a clinical germline and tumor WES study as well as optional procedures such as genomic data sharing independent of race, ethnicity or language spoken.

**Electronic supplementary material:**

The online version of this article (doi:10.1186/s13073-014-0069-3) contains supplementary material, which is available to authorized users.

## Background

The process of obtaining informed consent for subject participation in clinical research protocols is multifaceted and includes the researcher and subject or guardian reviewing the study purpose, procedures and informed consent document. Previous studies investigating parental and young adult preferences for informed consent in clinical trials in pediatric oncology settings have demonstrated that participants generally prefer: (1) to receive more information but risk feeling overwhelmed with the quantity or pace of information provided; (2) that the information be presented in a stepwise and organized manner; and (3) to be given sufficient time to process the information, especially in the context of their emotional state, before making a decision [1,2]. The presentation of additional audio or visual learning materials is also suggested [1]. These studies emphasize that, while there are essential aspects of informed consent, details need to be adapted to fit the needs and questions of the individual or family.

The challenge of successfully informing potential participants without overwhelming them with content can be particularly daunting for research involving genome-scale tests, given the amount and complexity of the information to be conveyed and the differential informational priorities and preferences of the parties involved (patients, parents, clinicians) [3]. It is critical that the participant understand the purpose of the research, the type of test(s) to be performed, the various types of results that they will receive (and not receive) through participation and in which situations they have a choice about receipt of results in these categories, as well as the potential risks and benefits of participation. Studies have shown that, although the public is familiar with the terminology of genetics and its association with disease, there are still significant gaps in conceptual knowledge [4-7]. Although there is limited research looking specifically at public knowledge of concepts in the setting of whole exome sequencing (WES) and whole genome sequencing (WGS), early studies have illustrated such gaps exist but are improved by the informed consent process [8]. In addition, the long-term risks of the inclusion of genomic information in the medical record and research databases remain unknown, although some level of protective legislation is in place (through the Genetic Information Nondiscrimination Act of 2008, GINA) [9]. Thus, specific considerations for the informed consent process in studies utilizing WES/WGS include the higher likelihood of obtaining unanticipated results and the risk of identifiability or loss of privacy through data sharing [10]. As is true for other types of research, traditional barriers such as language and education level can also factor into the challenges of obtaining informed consent for WES/WGS studies. Previous studies have suggested a differential enthusiasm for participation in medical research among racial and ethnic groups [11-13], but further evaluation in the specific context of clinical genomic research is needed.

Patient understanding of genomic testing in cancer patients may also be complicated by confusion over the conceptual differences between germline mutations and tumor or somatic mutations. Interview studies assessing attitudes among adults diagnosed with cancer toward personalized medicine revealed that a majority of participants expressed understanding of the difference between germline and somatic genetic testing based on descriptions provided to them. However, when asked about specific benefits and risks of somatic testing some participants described examples typically associated with germline genetic testing, such as learning about familial risk or insurance discrimination, suggesting the distinction still may have remained unclear [14].

As WES and WGS are transitioned from the laboratory to the clinic, there is a need for additional research to provide insight into the best practices for obtaining informed consent for these tests in a variety of clinical settings. The BASIC3 (Baylor Advancing Sequencing in Childhood Cancer Care) study seeks to integrate information from clinical germline and tumor WES into the care of newly diagnosed solid tumor patients at Texas Children’s Cancer Center (TCCC) and evaluate the impact of these exome data on the patients’ families and oncologists as part of the National Human Genome Research Institute Clinical Sequencing Exploratory Research (CSER) program [15]. This study offers a unique opportunity to investigate the informed consent process for clinical germline and tumor WES in the pediatric oncology setting. The TCCC serves a racially and ethnically diverse patient population, including a large number of families of Hispanic ethnicity and frequently utilizing Spanish-speaking interpreters and Spanish consent forms for both clinical care and enrollment on research studies, facilitating evaluation of the role of such factors in the informed consent process. The goals of this manuscript are to describe the informed consent process utilized for BASIC3 and to report on our experience for the first 100 families enrolled, including (1) the proportion of parents who decline enrollment of their child on this clinical WES study and their reasons for doing so, (2) a comparison of clinical and demographic factors (such as race and ethnicity) between patients whose parents agree to or decline study enrollment, and (3) the preferences of parents for participation in optional genomics study procedures such as inclusion of recessive carrier data in WES reports and deposition of patient data into research databases.

## Methods

### Study design

The BASIC3 study was approved by the institutional review board (IRB) of Baylor College of Medicine (BCM), which is also the IRB for Texas Children’s Hospital (TCH), the study clinical site. The study conformed to the Declaration of Helsinki. Study enrollment is offered for all patients with newly diagnosed solid tumors (including central nervous system (CNS) tumors) under the age of 18 years who undergo their initial tumor surgery and have ongoing oncologic treatment at TCCC and have at least one parent who speaks English or Spanish. Enrollment for parent-specific study procedures is separately offered to the parents of the patients. All study documents, education aids and consent forms have been fully translated into Spanish and medically trained Spanish interpreters are utilized as needed. The primary oncologists caring for the BASIC3 patients are separately consented to participate in study procedures, including oncologist interviews and surveys, but that process is not described in detail here.

**Figure 1:**
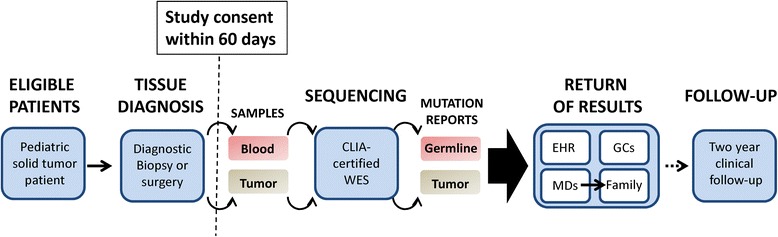
**BASIC3 clinical study design.** CLIA, Clinical Laboratory Improvement Amendments; EHR, electronic health record; GCs, genetic counselors; MDs, pediatric oncologists; WES, whole exome sequencing.

Patient-participants are enrolled in the study within 60 days of completion of the pathology report from their diagnostic cancer surgery (Figure 1). Newly diagnosed solid tumor patients are identified by the study team in cooperation with the TCH surgical and neurosurgical services, TCCC Solid Tumor and Neuro-Oncology clinical teams, and TCH pathology. Potential eligibility is confirmed through a review of the medical record and in consultation with the primary oncologist.

In brief, the study procedures are as follows (Figure 1). After informed consent for study enrollment (described in detail below) has been obtained, patient blood and tumor samples are sent to the Clinical Laboratory Improvement Amendments-certified and College of American Pathologists-certified Whole Genome and Cancer Genetics Laboratories of the Medical Genetics Laboratories of BCM for germline (including the mitochondrial DNA) and tumor WES. If tumor sample is not available for research, patients remain eligible for the study but only germline WES is performed. Blood samples are obtained from both biological parents when available and utilized by the laboratory to interpret the inheritance of germline variants identified by WES in the patient-participant as previously described [16]; of note, these parental samples are not subjected to clinical WES. After the germline and tumor WES reports are generated (turnaround time of 3 to 4 months), they are placed into the electronic health record and reviewed with the patient’s primary oncologist by the BASIC3 principal investigators and genetic counselor(s).

**Figure 2:**
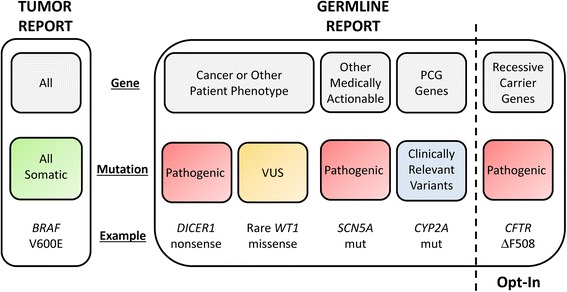
**Categories of whole exome sequencing results returned to BASIC3 study families.** PCG, pharmacogenetic; VUS, variant of uncertain significance.

The focused germline WES reports, as previously described [16], include all variants identified in four categories (Figure 2): (1) deleterious mutations in disease genes related to cancer susceptibility or other patient phenotype; (2) variants of uncertain significance in those same phenotype-associated disease genes; (3) medically actionable mutations in disease genes unrelated to cancer susceptibility; and (4) limited panel of pharmacogenomic variant alleles. In addition, at the time of study enrollment parents (or the child’s legal guardian(s), hereafter referred to as parents) are given the option to have the report include their child’s carrier status results for recessive disease. The germline reports utilized for this study do not include other disease-associated mutations, including those associated with adult-onset conditions unrelated to cancer. The tumor WES reports (Figure 2) comprise an annotated listing of all somatic (tumor-specific) mutations identified in the patient’s tumor, prioritized based upon potential clinical relevance for the patient-participant.

After the parents have been notified that their child’s WES results are available, an appointment is scheduled for a ‘disclosure visit’ to review the results with their primary oncologist and a study genetic counselor in the TCCC clinic or inpatient oncology unit. A hard copy of the WES report(s) and a genetic counseling letter are provided to the family at this visit, which is audiorecorded. The longitudinal aims of this study include one parent per family being asked to complete surveys and a subset being interviewed at three time points: after study enrollment, after receipt of WES results, and one year after receipt of WES results.

### Informed consent process

The study team actively involved in the enrollment process includes SEP, study PI and clinical geneticist; DWP, study PI and pediatric oncologist; SS and KB, study genetic counselors; RK, study coordinator. The patient’s primary pediatric oncologist advises the study team on when an appropriate time to meet with the family might be, given the clinical status of the patient and the emotional state of the family. Once this ‘green light’ has been given by the oncologist, one or more members of the study team approaches the family about the study. First, a brief (<10 minutes) overview of the study is provided by the BASIC3 team and a study brochure is provided. For families who express interest after the study overview, a more detailed explanation of the study is provided, referred to here as the consent conference. The setting for the consent conference is dictated by the clinical status of the patient and the preferences of the family. The information provided includes a full description of study events (Figure 1), the types of potential tumor and germline results reported (Figure 2), and the risks and benefits of study participation. As background for understanding the study, a brief ‘genetics lesson’ is provided for the families, including concepts such as how cancers develop and the distinctions between tumor (somatic) and germline mutations. Ideally, both parents participate in this initial conference; however, often only one parent is available and the ultimate decision about study enrollment is delayed until the family can discuss the study at home and/or a follow-up conference with the second parent is held.

Several printed visual aids (in English or Spanish) are used to complement the informed consent document and verbal overview. First, as noted above, a study brochure is provided to parents either by the oncologist or during the consent conference which includes a brief introduction to the study goals and procedures, an overview of the relevance of germline and somatic mutations to cancer development (with two diagrams), and a listing of the types of potential results that may be received during the study. Second, bulleted printed summaries of the types of results that may be received in both the tumor and germline reports are provided to emphasize this important information. Finally, aids are used to explain the concepts of carrier status for recessive and X-linked conditions. These consist of publicly available diagrams published by the US National Library of Medicine [17,18] as well as key facts (developed by the study staff) regarding each mode of inheritance, including what it means to be a carrier, how this information applies directly to the participant and other family members, and reproductive risk.

### Study consent forms

Separate consent forms are used for the enrollment of patients and their parents in the study. The Patient Consent Form (Additional file 1) on which the parent consents to the enrollment of their child in the study describes all key study-related events (Figure 1), including the study samples to be obtained and the details of the germline and tumor WES tests. The purpose of the study is explained as learning ‘how to best report and use the clinical exome sequencing test results for childhood cancer patients.’ Specific examples of the types of potential clinically relevant tumor WES results and the likelihood of such an event are described, such as ‘rare’ cases in which tumor findings might include mutations that ‘are normally found in a different type of tumor from what your child was diagnosed with’ (for example, mutations of potential diagnostic utility) and ‘very rare’ cases in which the mutations ‘make your doctor think your child’s tumor will respond better or worse to a particular cancer treatment’ (for example, mutations of potential therapeutic utility). Similarly, categories and examples of potential clinically relevant germline WES results are provided (Figure 2), including ‘inherited mutations that cause your child to have an increased risk of developing other cancers…[and] may also provide information about the risk of cancer in close family members’ (for example, cancer susceptibility mutations), ‘inherited mutations that are unrelated to cancer but provide information about a different medical condition for which treatment is available and recommended as standard medical care’ (for example, medically actionable incidental findings), and ‘inherited mutations that might affect how your child’s body responds to certain medicines’ (for example, pharmacogenomic variants).

In addition to the required study procedures, the Patient Consent Form also includes a number of optional elements to which parents may separately consent or decline, including the option to include or exclude the child’s recessive carrier mutations in the germline WES report. Other choices relate to additional research procedures, such as the analysis of patient samples using other non-clinical genomic methods and the release of the child’s genetic and clinical information into scientific databases (Table 1).

**Table 1:** **Optional BASIC3 study events listed in patient and parent consent forms**

**Participant**	**Study event**
Patient	Inclusion of carrier status results in germline exome report
Patient	Use of diagnostic tumor sample for additional genomic research
Patient	Use of blood sample for additional genomic research
Patient	Use of future tumor samples for additional genomic research
Patient	Release of blood/tumor samples and genetic/clinical information to other researchers
Patient	Release of genetic and clinical information into scientific databases
Parent	Use of blood sample for additional genomic research
Parent	Release of blood sample and genetic/clinical information to other researchers
Parent	Release of genetic/clinical information into scientific databases
Both	Permission for future re-contact

Several potential risks of study enrollment are included in the consent form, including the physical risks of obtaining a blood sample, the risk of anxiety related to disclosure of genetic results, and the potential disclosure of non-paternity through genetic testing. The risk of loss of privacy of genetic information is discussed in relation to the collection and storage of research data as well as the inclusion of genetic information in the medical record, for example, ‘Health insurance companies may also have access to this information. There are laws to protect against the use of this information in making decisions about health insurance and employment. However, you may be asked to provide medical record information when your child applies for life insurance or disability insurance’. It is stated that there are ‘additional risks of loss of privacy’ if parents consent to the optional study procedures of sharing genetic and clinical data with other BCM investigators or the de-identified release of genetic information into scientific databases.

The possibility of identifying either tumor or germline data that may impact the care of the patient and/or other family members is listed as a potential benefit; however, it is stated that ‘we do not think that the mutations that are found are likely to change the planned cancer treatment for most children’ and that in that circumstance the benefit is more to society than the individual. The probability of a tumor mutation being identified that has implications for treatment is estimated to be ‘very rare’. It is clarified that there are no study costs for patients and that families will be reimbursed a nominal amount ($25) for participation in study surveys and interviews.

For parents who decline participation for their children we record the answer to an open-ended question: ‘Would you please tell us why you decided to not participate’, patient tumor type, age at diagnosis, language used during the consent conference (English or Spanish), use of an interpreter for consent conference, and race/ethnicity as listed in the electronic health record at the time of initial hospital admission.

The Parent Consent Form (Additional file 1) on which the parent consents to their own enrollment in the study utilizes similar structure and language to the Patient Consent Form, including identical Background and Purpose sections as well as a description of potential WES results and study risks and benefits. Information about required and optional study samples and procedures for the patient is replaced by a description of optional parental blood samples that may be provided for interpretation of variants identified in the patient’s germline WES report and additional research procedures. No parental blood samples are required for study participation.

### Child assent for study enrollment

As per BCM and TCCC guidelines, assent was obtained for each child who was judged ‘capable of providing assent based on age maturity and psychologic state’, and documented by the parent on the Patient Consent Form document using the standard BCM/TCCC language (Additional file 1). TCCC standard operating procedures for obtaining informed consent for research studies recommends that assent be obtained from subjects greater than 6 years of age and under 18 years of age. The extent of child participation in the consent process is guided by each child and their parents. The consent conference(s) is conducted with the child in the room and the child is encouraged to participate to the extent of their interest and understanding. In only one case did the parents specifically request to have their child not be present for part of the discussion: a 12 year old girl with a CNS tumor and developmental delay who was present for most of the conference but became bothered by the lengthy discussion of the study.

### Data collection and analysis

A password-protected web accessible study database was created to track all study events and procedures and collect clinical data for enrolled subjects, including subject age, gender, race, ethnicity, tumor diagnosis, grade, stage, the presence of metastatic disease at diagnosis, and whether chemotherapy and/or radiation therapy was planned.

Subjects’ characteristics were summarized descriptively. Comparisons were made between enrolled and non-enrolled patients for the following variables: patient age, gender, ethnicity, race, tumor location (CNS or non-CNS), whether an interpreter and Spanish consent form were used, whether tumor was available for WES, and the time from surgery to the decision about study enrollment. Two sample rank sum tests were used to compare the continuous data and Fisher’s exact tests were used for the categorical data. The *P*-value for ethnicity was calculated with ‘not reported’ subjects excluded. For race, the comparison made was between ‘white’ subjects and all others with ‘not reported’ subjects excluded.

## Results

The study staff began approaching all families of potentially eligible newly diagnosed solid tumor patients on 1 August 2012 and the one-hundredth subject was enrolled on 3 September 2013. Over this time period, 121 families met eligibility criteria and were offered enrollment in the study. Twenty-one families declined enrollment, resulting in a rate of study enrollment of 83% (100/121, 95% confidence interval 75% to 88%). Due to the complexity of the study and the potential implications of study enrollment for other family members, the study consent conference is generally conducted as a multi-step process rather than a single meeting, with most occurring in the patient room on the inpatient oncology floor (if the child is hospitalized) or in the outpatient TCCC clinic, often while patients are having chemotherapy infusions. The interval from date of initial tumor surgery to parental decision about study enrollment ranged from 5 to 63 days (median 36 days) for patients enrolled in the study. Enrolled patients were diagnosed with a diverse representation of pediatric solid tumors. Tumor was available for WES in 84% of subjects. Further tumor-directed treatment (chemotherapy and/or radiation therapy) was planned for 82% of subjects. Initial evaluation revealed that 34% of tumors were metastatic at the time of diagnosis.

**Figure 3:**
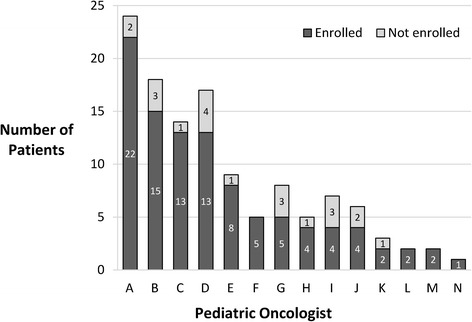
**Patient enrollment by primary oncologist.**

Forty-five percent of enrolled subjects were female (Table 2). Ages ranged between 1 month and 17 years (median of 5.1 years) at the time of tumor surgery. Forty-three percent self-identified as being of Hispanic ethnicity and 56% as white race. A Spanish-speaking interpreter and Spanish consent form were utilized for 15% of subjects. Fourteen different oncologists had patients enrolled in the study, with the number of enrolled patients per oncologist ranging between 1 and 22 (median 4.5, mean 7.1) and the fraction of eligible patients per oncologist enrolling in the study ranging between 57% and 100% (Figure 3). There was no correlation between the number of patients approached per oncologist and the fraction enrolled (Spearman’s rank correlation = -0.35, *P* =0.21).

**Table 2:** **Characteristics of patients enrolled and not enrolled in the study**

**Characteristic**		**Enrolled (n = 100)**	**Declined (n = 21)**	***P*** **-value**
Median age in years (range)		5.1 (0.1-17.0)	4.0 (0.1-14.2)	0.73
Female gender		45 (45%)	5 (24%)	0.09
Ethnicity				1^a^
	Hispanic	43 (43%)	10 (48%)	
	Non-Hispanic	51 (51%)	11 (52%)	
	Not reported	6 (6%)	-	
Race				0.17^b^
	White	56 (56%)	18 (85%)	
	Black or African American	12 (12%)	1 (5%)	
	Asian	4 (4%)	1 (5%)	
	American Indian or Alaska Native	4 (4%)	1 (5%)	
	Multiple	6 (6%)	-	
	Not reported	18 (18%)	-	
Tumor location				0.22
	CNS	33 (33%)	10 (48%)	
	Non-CNS	67 (67%)	11 (52%)	
Tumor metastatic at diagnosis		34 (34%)	ND	
Adjuvant tumor treatment planned		82 (82%)	ND	
Tumor available for WES		84 (84%)	14 (67%)	0.12
Interpreter and Spanish consent form used		15 (15%)	4 (19%)	0.74
Median days from surgery to study decision (range)		36 (5-63)	42 (20-61)	0.052

^a^ *P*-value was calculated with ‘Not reported’ subjects excluded. ^b^Comparison was made between ‘White’ versus others with ‘Not reported’ subjects excluded. Two sample rank sum tests were used to compare the continuous data and Fisher’s exact tests were used for the categorical data. CNS, central nervous system; ND, not determined; WES, whole exome sequencing.

**Figure 4:**
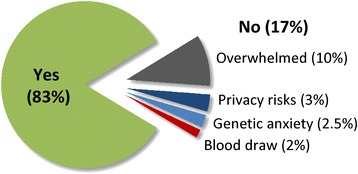
**Proportion of patients enrolled in the study and stated reasons for parents declining enrollment.**

The most common reason provided by parents for choosing not to participate in the study was that they were overwhelmed by their child’s new cancer diagnosis and did not wish to participate in an additional research study (10% of families offered study enrollment; Figure 4). Other reasons reported included concern about privacy risks (3%), anxiety about receiving genetic testing results (2.5%), and a desire for no further blood to be obtained for research study procedures (2%).

No significant differences were detected when comparing the 100 enrolled patients with the 21 patients who were not enrolled in the study for age, gender, tumor location (CNS or non-CNS), whether tumor was available for WES, or the time from surgery to the decision about study enrollment (Table 2). In addition, no significant difference was observed in the proportion of children identified by their parents as being of Hispanic ethnicity between those who enrolled in the study (46% of patients with race reported) compared with those who declined enrollment (48%). Similarly, a Spanish interpreter and Spanish consent forms were used in a similar proportion of parents agreeing to (15%) or declining study enrollment (19%). The proportion of patients identified as being of white race was numerically but not significantly lower for children enrolled in the study (68%) compared with those not enrolled (85%, *P* =0.17).

**Figure 5:**
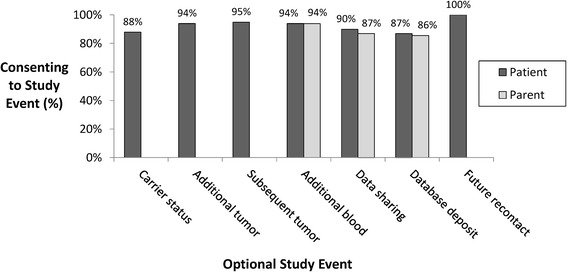
**Consent for optional study events.** Carrier status: reporting of germline carrier status for recessive diseases. Additional tumor: use of diagnostic tumor for additional research studies. Subsequent tumor: use of tumor from subsequent surgeries for additional research studies. Additional blood: collection of blood sample for additional research studies. Data sharing: sharing of study samples and/or de-identified genetic/clinical data with other investigators for IRB-approved studies. Database deposit: deposition of de-identified genetic/clinical data into scientific databases. Future re-contact: to obtain follow-up clinical information or request additional study samples.

We then examined decisions about optional patient-related study procedures made by the parents of the enrolled children (Figure 5). Notably, 88% of parents chose to have information about carrier status for recessive diseases included in their child’s germline WES report. Parents also consented to the remaining optional study procedures at a very high rate, including use of the diagnostic tumor sample for additional research studies (94%), collection of tumor for research purposes from any subsequent tumor surgeries (95%), collection of an additional 1 to 2 teaspoon blood sample for research purposes (94%), sharing of de-identified genetic and clinical data with other BCM investigators with IRB-approved protocols (90%), and deposition of de-identified genetic and clinical data into scientific databases (87%). All parents allowed future re-contact by study investigators.

One hundred and forty-seven parents (1.5 parents/patient) chose to enroll in the study, resulting in 4 families with no parent enrolled, 45 with one parent enrolled, and 51 with two parents enrolled. Two enrolled parents were not biologically related to the enrolled child and were not asked to provide blood samples for the study. Most parents consented to the optional study procedures (Figure 5), including collection of an additional 2 teaspoon parental blood sample for research purposes (94%), sharing of de-identified genetic and clinical data with other BCM investigators with IRB-approved protocols (87%), and deposition of de-identified genetic and clinical data into scientific databases (87%). All enrolled parents allowed future re-contact by study investigators. With regard to actual phlebotomy data, blood samples for interpretation of their child’s germline WES results have been obtained to date from 68% of enrolled parents; blood samples for additional research have been obtained from 67% of parents consenting to that optional study procedure.

## Discussion

Parents of children with newly diagnosed cancers were found to have significant interest in a research study of clinical and tumor WES, with more than 80% agreeing to study enrollment. No significant differences were seen in the clinical and demographic factors analyzed between the cohorts of patients enrolled and not enrolled on the study, including race, ethnicity, the use of Spanish interpreters and consent forms and the type of tumor diagnosed. In this clinical setting it is perhaps unsurprising that parents would be eager to obtain any potentially clinically relevant information that might help guide treatment of their child’s cancer, even if it is described as being of uncertain benefit by study investigators. This is consistent with previous studies which demonstrated that the majority of mothers of childhood cancer patients were willing to have their child undergo genetic testing for cancer susceptibility at a time when the benefits of such testing remained hypothetical [19]. However, two observations suggest that factors other than a desire for tumor WES results that could direct cancer therapy also play a role in the decision of parents to enroll in the BASIC3 study. First, most parents of patients for whom no tumor was available for WES still consented to study enrollment, albeit at a slightly decreased rate: 70% if no tumor compared with 86% if tumor available (*P* =0.12). This could result from the fact that our study provided germline WES on study patients. Questions such as ‘why did this happen to my child?’ and ‘will my other children also get cancer?’ are a nearly universal component of initial diagnostic discussions between oncologists and families of newly diagnosed cancer patients. Germline WES of their child has the potential to provide insight into these questions by identifying pathogenic cancer susceptibility mutations. Perhaps equally importantly for families, it may help to provide reassurance that the patient’s siblings are unlikely to develop a childhood cancer if no such mutations are found. Second, a very high proportion of parents of children enrolled in the study also agreed to optional study procedures that have both immediate risks (blood draws) and theoretical risks (privacy and insurability concerns) despite the fact that those procedures were not required to obtain either tumor and germline WES results, indicating that altruistic considerations were also likely relevant to decision-making by parents regarding enrollment in this study of clinical genomics.

Although not emphasized in discussions of genomic sequencing, the clinical context is very important to the consent process [20]. The period surrounding a new diagnosis of cancer or other life-threatening disease is one of remarkable stress for patients and their families, which can complicate the informed consent process [21,22]. Accordingly, we attempted to be sensitive to the specific clinical and social circumstances when approaching families about potential study enrollment, including close coordination with the primary oncologist and inpatient oncology team for determination of the timing and ideal setting (inpatient or outpatient) for this initial contact. For example, several months after the study opened, we extended the allowable window for study enrollment from 30 days to 60 days from definitive pathologic diagnosis in order to provide additional flexibility in the consent process. Despite these efforts, the reason provided by the majority of parents who chose not to enroll their child in the study was that they were overwhelmed by their child’s new cancer diagnosis and treatment and did not wish to participate in any research study. Consequently, the observed 17% rate of families declining enrollment in the study may overestimate the proportion of parents who would decline WES in a clinical, non-study setting not requiring a long study consent process or additional research study procedures. Given that their child’s illness was the most frequent explanation provided by parents for declining study enrollment, it is possible that the children whose parents declined were more severely ill than those who were enrolled in study. We did not obtain detailed clinical data on the non-enrolled children and cannot compare the clinical characteristics of the two groups; however, the diagnoses of the non-enrolled patients (including five pilocytic astrocytomas and three Wilms tumors, both tumor types with excellent survival rates) do not appear to carry an unusually poor prognosis. Parental reasons for declining enrollment related to the anticipated risks of clinical sequencing described in the consent form, such as the loss of privacy and potential anxiety about genetic test results, were in aggregate reported by only 5.5% of parents approached (for example, by 33% of parents declining study participation).

A majority of the time during study consent conferences was spent discussing the types of results that can be discovered by tumor and germline WES (and the potential clinical relevance of germline findings for other family members) and the risks of genomic research, with a particular emphasis on the implications of the loss of privacy of genomic data. It was stressed to parents that tumor and germline WES reports for patient-participants in the study would be incorporated into the electronic health record and that families should not participate in the study if they objected to that required study procedure. The finding that more than 80% of parents elected to enroll their children in the study suggests that the perceived risks of genomic research were outweighed for most families by the potential benefits of study participation. This concept has been previously described among parents offered genome scale research sequencing (where results did not appear in the medical record) in the setting of chronically ill children [23]. Overall, the clinical context of the genomic study appears to play a role in the potential subjects’ willingness to participate despite potential concerns about obtaining genetic information. In the context of a child recently diagnosed with a life-threatening condition such as cancer, parents were generally very interested in participating in the study and only a small percentage viewed these potential ‘genetic privacy’ risks as reason enough to decline participation. The observed rate of enrollment in this genomic study mirrors the high participation rates on therapeutic clinical trials for children with cancer [24].

Our study has several key limitations which offer opportunities for future research. First, the data reported do not include an assessment of parental understanding of the study procedures or the key concepts of genetics and genomic sequencing. The extent to which parents comprehend the complex issues inherent in clinical WES when they consent to their child’s participation (and are not simply agreeing to participate in the hope that clinically relevant information can be obtained) and whether differences in understanding exist between the parents who agree or decline to have their child participate in the study remain unclear. Given previous research suggesting that participants in both cancer trials [25] and genomic studies [26] often have a poor understanding of relevant scientific concepts and study procedures, further study of this topic is needed. Data to be obtained through longitudinal surveys and interviews of BASIC3 study parents may provide relevant information regarding parental understanding of study procedures and level of satisfaction with study enrollment. Second, relatively little information (consisting of a brief explanation of reasons for declining study participation and limited clinical and demographic data) was collected from parents who declined participation on the study. A more extensive analysis of study ‘decliners’ would have the potential to provide deeper insight into the factors behind this decision and could be useful for improving the informed consent process, although it would be potentially difficult to obtain since this group of parents stated that they were currently overwhelmed with their obligations. Finally, although the childhood cancer patients were present for the informed consent conferences and included (to the extent of their interest and capabilities) in study discussions, the current study does not provide data on the details of their involvement or their understanding of study procedures. Further research will be required to shed light on this critical aspect of clinical genomic studies involving pediatric patients.

## Conclusions

Obtaining informed consent for clinical tumor and germline WES of children with newly diagnosed cancers offers many challenges, most fundamentally the difficulty of conveying complex genomic information in sufficient, but not excessive, detail to families who are immersed in a medically and personally critical situation. We have developed an informed consent process and document for clinical WES in the pediatric oncology clinic that utilize a dedicated informed consent team with specific genomics training and experience and rely upon active communication with the patient’s clinical team regarding the clinical status of the patient and the emotional status of the family. As is the case when obtaining consent for treatment protocols for newly diagnosed patients, flexibility in all aspects of the informed consent process is critical at this unpredictable and stressful time for families [1,27], including the timing and location of the initial conference as well as the understanding that the ‘conference’ is in reality most often a series of meetings involving multiple family members and significant repetition of information, consistent with the concept of informed consent as a process and not a single event [28]. Although we did not collect data on the duration of the consent conferences, we estimate that the typical time per family is approximately an hour, similar to that required to consent families to cancer treatment protocols [29,30]. Key points in our consent forms (Additional file 1) and the consent conference(s) include: an explanation of the multiple different types of potential tumor and germline WES results, with specific examples of mutations in each category provided; an emphasis on the fact that germline results have potential implications for other family members; and careful explanation of the known and unknown privacy risks of having genomic information in the medical record, including what is protected (and not protected) by GINA and full translation of all consent documents and educational aids for non-English speaking families.

Most parents of children with newly diagnosed cancers were willing to allow clinical tumor and germline WES to be performed, as well as optional research procedures without the possibility of direct benefit to the child or family. In this clinical setting, we did not observe that the willingness to participate in clinical genomics research was significantly impacted by patient-specific factors such as age, gender, race, or ethnicity. It is our impression that the parents of most children with newly diagnosed cancers are singularly focused on their child’s diagnosis and clinical care and their assessment is that the potential benefit of identifying any clinically relevant information (even if unlikely) through WES outweighs risks of privacy loss and genetic anxiety. Follow-up study data, including ongoing analysis of semi-qualitative longitudinal interviews with study parents (before and after receipt of WES results as well as one year later), will help to shed light on this decision-making and differences in parental perspectives on clinical WES over time.

## Additional file

Additional file 1:
**Patient and Parent Consent Forms for the BASIC3 study.**

## Abbreviations

BASIC3: Baylor Advancing Sequencing in Childhood Cancer Care
BCM: Baylor College of Medicine
CNS: central nervous system
GINA: Genetic Information Nondiscrimination Act
IRB: institutional review board
TCCC: Texas Children’s Cancer center
TCH: Texas Children’s Hospital
WES: whole exome sequencing
WGS: whole genome sequencing
