# Supplementary material for: Obtaining informed consent for clinical tumor and germline exome sequencing of newly diagnosed childhood cancer patients
Source: Genome Med. 2014 Sep 17;6(9):69. doi: 10.1186/s13073-014-0069-3 (PMC4195891; doi:10.1186/s13073-014-0069-3)
Supplement: Additional file 1: — Patient and Parent Consent Forms for the BASIC3 study. [file 13073_2014_69_MOESM1_ESM.pdf]

INCORPORATION OF GENOMIC SEQUENCING INTO PEDIATRIC CANCER CARE

Background

Your child is being invited to participate in a research study. Your child's participation in this study is entirely voluntary. Should you choose to have your child withdraw from this study, your decision will in no way affect the care that your child receives. Please read the consent form carefully and feel free to ask any questions before you agree to take part in the study. You will be given a copy of the consent form to keep if you decide to participate in the study.

Cancer is a disease caused by changes (mutations) in the genetic code of a cell. The genetic code is like a set of instructions that tell our cells how to grow properly. These instructions are referred to as genes. The mutations in genes found in cancer cells cause cells to grow and spread abnormally. Sometimes one of these mutations is present in every cell of the body ("inherited mutations") and can be found in a blood sample, while others are only found in the cancer cells ("tumor mutations"). A clinical test is now available to look for certain kinds of mutations in all 20,000 genes in a cell. This test is called "exome sequencing." It is important to understand that exome sequencing cannot find all types of mutations that might occur in your child's tumor or blood.

In this study we will use exome sequencing to look for inherited and tumor mutations occurring in your child. The exome sequencing test is not experimental and is performed in a clinically certified laboratory. However, most children with cancer do not have it done as part of their regular cancer care, and no one knows the best way to use the tests for cancer patients yet.

Purpose

The main goal of this study is to learn how to best report and use the clinical exome sequencing test results for childhood cancer patients at the Texas Children's Cancer Center (TCCC). We plan to study how these results can best be explained to physicians and parents of cancer patients. We want to learn what both groups prefer in receiving the results. We want to learn whether the results can help us to: (1) better understand the future cancer risk of childhood cancer patients and their family members so that better methods of screening and prevention can be developed and (2) make treatment decisions for our patients if their cancer returns.

A second goal of this study is to learn more about the biology of childhood cancer and develop better ways to prevent, detect, and treat these cancers. To complete this goal we will use additional research methods to find new inherited mutations and tumor mutations occurring in childhood cancers and we will combine these results with clinical information from patients' medical records. Since this is not the main goal of the study, you will be asked whether you want to participate in these additional studies.

Procedures

COLLECTION OF STUDY SAMPLES

We are asking for your permission to use tumor and blood samples from your child. These will be obtained at (or near) the time of your child's tumor surgery and sent to the Baylor College of Medicine Whole Genome Laboratory (WGL) for exome sequencing. In addition, we request blood samples from both parents (if available) so that the WGL can confirm inherited mutations identified in your child. The WGL handles your samples the way any regular medical samples are handled, following all standard procedures for sample tracking, storage, and confidentiality.

1. Patient tumor sample. This sample is collected to find the tumor mutations using exome sequencing. Only tumor tissue that has been removed (or will be removed) as part of your child's routine diagnosis and treatment and is left over after all necessary clinical tests have been completed will be used for this study. No extra surgery will be done.

2. Patient blood sample. This sample is collected to find the inherited mutations using exome sequencing. We plan to obtain 1-2 teaspoons of blood. We will try to do this at the same time as other clinical tests are being ordered and/or by obtaining the blood sample through your child's central line (a long-term IV line). It is possible that an extra blood draw will be done.

3. Parent blood sample(s). These samples are routinely requested by the WGL as part of your child's clinical exome sequencing test in order to confirm the inherited mutations found in your child. The parent blood samples are not required for enrollment on the study and do not undergo exome sequencing as part of your child's clinical test. Both parents (if available) will be asked to donate 2 teaspoons of blood. This blood draw will be performed either in the TCCC clinic or at the General Clinical Research Center within one month of study enrollment at a time arranged with you.

WILL I GET TO SEE THE RESULTS OF GENETIC TESTING?

Clinical Exome Test Results:

The exome test takes a long time to finish. We think it will be about 3 months after we take the sample until the results are ready. The results of the exome test will be given to your child's cancer doctor and also placed into your child's electronic medical record. Before your child's doctor meets with you to review the results, he or she will be able to discuss them with the investigators on this study who are experts in exome tests. Your child's cancer doctor will then explain the results to you and work with you to decide whether anything about your child's care should change based on the results. A genetic counselor will work with your cancer doctor to explain the inherited mutations to you. These results will be explained to you in one or two meetings scheduled at the same time as your child's regular clinic visits whenever possible.

These tests are very new and we do not know yet how to use exome testing to guide children's cancer treatment. Because the test takes so long to do, the results will not be available until after your child begins his or her treatment. We do not expect the test results to change anything your doctor planned to do for treatment. Therefore we do not think that getting the results is likely to make your child's treatment better or make it more likely for him or her to be cured. It is possible, however, that the results might reveal tumor or inherited mutations that matter to the clinical care of your child or family such as:

1. Tumor mutations that are normally found in a different type of tumor from what your child was diagnosed with. We think this will be rare, but if it does happen, your doctor may talk to you about changing your child's treatment plan.

2. Tumor mutations that make your doctor think your child's tumor will respond better or worse to a particular cancer treatment. We think this will be very rare for most children. It might be more common in cases where a child's tumor has returned after standard treatment and additional treatment options are being considered.

3. Inherited mutations that might affect how your child's body responds to certain medicines. This information might help your child's doctors choose the dose of some of your child's medicines.

4. Inherited mutations that cause your child to have an increased risk of developing other cancers (or a second cancer of the same type). This information may also provide information about the risk of cancer in close family members. For example, children and adults who inherit mutations in the gene called p53 are at increased risk for multiple different types of cancer. If an inherited mutation in this gene is found in your child then additional cancer screening would be recommended and close family members should also be checked for the mutation so that they can be screened as well if necessary.

5. Inherited mutations that are unrelated to cancer but provide information about a different medical condition for which treatment is available and recommended as standard medical care. For example, we might find a child who inherited a mutation that puts him/her at high risk of life-threatening bleeding from a blood vessel (called an aneurysm). If that type of mutation were found we would recommend additional follow-up testing and/or treatment as part of standard medical care.

If we find any of these kinds of mutations, your child's cancer doctor and a genetic counselor will explain them to you and work with you to determine the most appropriate screening or treatment (if any) for your child and family members.

Exome sequencing can also reveal information about whether your child is a carrier of a genetic disorder such as Cystic Fibrosis. This information may not affect your child's health but it may be helpful to know later in life. Also, if a child has one of these kinds of mutations, he or she probably inherited it from a parent. Therefore the parents might want to be screened to see if there is a risk of having a child who has a genetic disorder. However, some people do not want to know this kind of information. Please initial below whether you would like this type of genetic information to be included in your child's exome sequencing report:

Yes_____ I would like to receive information about whether my child has inherited any mutations that indicate that they are a carrier of a genetic disorder.

No______ I would not like to receive this information and do not want it included in my child's exome sequencing report.

PROCEDURES TO HELP US LEARN THE BEST WAY TO COMMUNICATE CLINICAL EXOME TEST RESULTS

Because we want to learn more about how doctors and patients discuss exome test results, we will include the following activities if you participate in the study:

1. Audiorecording of clinic visit(s) where you learn about the results of exome sequencing. By studying how doctors and parents talk about genetic test results this will help us learn how to improve communication and understanding about these results between cancer doctors and patient families.

2. Parent surveys. Parent surveys will be conducted at three different time points during the study: soon after initial study enrollment, after disclosure of the exome sequencing results, and one year later. Each family will tell us which parent will fill out the survey at all three of the time points. The surveys will ask questions about your family history of cancer, your knowledge of genetics, how you like to learn

information from doctors and how you like to make decisions. The surveys will take approximately 30 minutes to complete at your convenience.

3. Parent interviews. Some families will be randomly selected for more in-depth interviews at three different time points during the study: soon after initial study enrollment, after disclosure of the exome sequencing results, and one year later. Each family will select one parent toparticipate in all three interviews. The interviews will ask more detailed questions about what you expect to learn in this study, what information you are most interested in learning and how you make decisions. Each interview will take place at TCH, last approximately 1 hour, and be audiorecorded. We will try to schedule these interviews at times that would be convenient to you.

4. Physician interviews. We will interview the cancer doctors to find out what they find most useful in the exome reports and what they think are the best ways to talk about this information with families. We will also collect information from your child's medical records, including their age, ethnic background, diagnosis, disease history, medical treatments, and response to treatments. We hope this will help us understand whether the exome results are related to how children with cancer do.

ADDITIONAL LABORATORY RESEARCH STUDIES

After the exome sequencing test has been performed on your child's tumor sample, there may be some sample left over. If so, we would like to do some additional genetic research tests. We are also requesting an additional blood sample that will be used for research. This may include using newer methods of sequencing, or sequencing parts of the DNA that doesn't contain genes or studying the RNA and proteins that are coded by your DNA as well as grow blood cells in the laboratory to test for gene function. We may also try to grow tumor or blood cells in the laboratory.

Any results of these research tests would be preliminary and would not be reported to you or placed in the medical record. If research from this project is presented at research conferences or published in professional journals, we will not use any information such as name, address, telephone number, or social security number, included in the presentations or publications. If we identify a genetic change that we think is clinically important, we will share those results with your child's cancer doctor so that they can discuss them with you and make follow-up plans. It is important to realize that these will be research results, and must be confirmed in a clinical laboratory in order to be used for clinical purposes.

Please initial below whether you agree to the use of your child's tumor and blood samples for these other genetic research tests:

Yes_____ No_____ I consent to have these leftover tumor samples used for additional genetic research tests.

Yes_____ No_____ I consent to a blood sample (1-2 teaspoons) from my child for additional genetic research tests.

If you have consented to have your child's samples submitted to the Texas Children's Cancer Center Tissue Bank (in a separate consent document), we will give any leftover samples to the Tissue Bank.

Similarly, we would like to do additional genetic research tests on parent blood samples and attempt to grow blood cells in the laboratory to use for tests of gene function. These tests could include exome

sequencing, which is otherwise not performed on the parent samples. Unlike your child's exome sequencing results, the results of these additional tests would be used for research purposes only and the results would not be reported back to you or placed in your or your child's medical record unless we identify a genetic change that we think is clinically important, in which case we will give you that information and explain how to have it confirmed by a clinical laboratory.

Patient tumor samples from additional tumor surgeries at TCH:

We would also like to collect research samples from any future surgeries that might occur so that we may study how tumor mutations change over time as tumors are treated. These samples are not required for enrollment on the study. Only tumor tissue that has been removed (or will be removed) as part of your child's routine diagnosis and treatment and is left over after all necessary clinical tests have been completed will be used for this study. No extra surgery will be done.

Please initial below if you consent to the collection of your child's tumor tissue from any future surgeries (if necessary) for research purposes:

Yes_____ No_____ I consent to the use of my child's tumor tissue from future surgeries.

If you agree to participate in this study, these biological samples will be obtained and used for research. This research material will not be available to use for other clinical testing.

Who will have access to your additional study information?

As we explained the clinical exome report will be handled like any other clinical test and placed in the medical record and explained to you by your physician. For the laboratory research studies, questionnaires, audiotapes and survey information, these results will also be stored in a confidential computer database along with all data about your child's biological samples and parents' biological samples (if provided). These biological samples and medical information will be labeled with a code, meaning that names and other identifying information are removed and samples are given computer-assigned numbers that the research team can use to access them. Only the investigators and selected research staff will be able to match the code to a particular person. Only the investigators and selected research staff will be able to access the database.

In order to speed research, other researchers would like to be able to study your child's blood and tumor samples and have access to your child's genetic information so that they can compare it to the genetic information of others from other research studies and use it to answer future research questions. This information is most valuable when it is linked to some information about your child's medical history (clinical information). It would also be helpful to use your blood samples and genetic and clinical information for this research. If you agree, de-identified research blood and leftover tumor samples, as well as de-identified parts of you and your child's genetic information, and in some instances, clinical information, can be shared with other researchers at Baylor College of Medicine (BCM) who are conducting approved research studies. In addition, you and your child's genetic and clinical information can be shared by releasing it into scientific databases including those maintained by BCM and some maintained by the National Institutes of Health. These databases are restricted and can only be accessed by approved researchers. Sharing this information will help advance medicine and medical research by allowing other researchers to use this information to help solve questions of what causes cancers and other diseases.

There is a risk that others will be able to trace this information back to your child, which may impact the ability of your child or other family members to obtain life insurance, health insurance, or other products that may take into account the result of these genetic studies. Nobody will be able to know just from looking at a database that the information belongs to your child. However, because your child's genetic information is unique, there is a small chance that someone could trace the information back to your child or close biological relatives. The current risk of this happening is very small, but may grow in the future as new ways of tracing the information back to you or your close biological relatives are developed. Thus, the risk that your privacy would be breached may increase over time. Researchers who access your genetic and clinical information will have a professional obligation to protect your privacy and maintain your confidentiality.

The decision of whether or not to allow genetic and clinical information about your child that will be gathered in this research into scientific databases is completely up to you. There will be no penalty to you if you decide not to allow this information to be used. You can still participate in the main part of the study without allowing your child's information to be released outside the study. We strongly encourage you to discuss the decision about de-identified data release and its associated risks for you and your biological relatives with your family before you decide whether to consent to this part of the study.

Please initial below whether you agree to the de-identified release of your child's research blood and leftover tumor samples and genetic and clinical information for other approved research studies at BCM and/or into scientific databases:

Yes_____ No_____ I consent to release of my child's research blood and leftover tumor samples and genetic and clinical information to other BCM researchers conducting approved research studies.

Yes_____ No_____ I consent to release of my child's genetic and clinical information into scientific databases.

Permission for future recontact by study investigators

We will follow each child in the study for 2 years to determine if their cancer doctor has found the exome information useful in their care. In the future, it may be helpful to our research to be able to recontact you to obtain additional clinical information or to ask your permission to collect another research sample. Please initial below if you we may recontact you after those 2 years are completed to discuss these research opportunities.

Yes_____ No_____ I consent to be contacted in the future for research study purposes.

Can I change my mind after I agree to let our samples be used?

You can withdraw from this study for any reason at any time. If you decide to withdraw from the study, your child's samples will be discarded. If you had agreed to let your child's samples be submitted to the TCCC Tissue Bank, you will be asked at the time of withdrawal from this study whether you would like these samples to be discarded or kept in the Tissue Bank for future research.

If you decide to withdraw from this study after your child's genetic code has been analyzed, your genetic information will be discarded and will not be used in this study. However, if your child's tumor and inherited exome sequencing reports have already been submitted into the medical record, it will not be possible to remove these reports from the medical record. In addition, if you agreed to have your child's genetic and clinical information shared with other investigators or released into scientific databases, it may not be possible to remove this data from the database.

You can see and get a copy of your research related health information. Your research doctor may be able to provide you with part of your information while the study is in progress and the rest of your information at the end of the study.

The only physical risk of this study is related to obtaining the blood sample. The risk of drawing blood includes a small risk of bleeding or infection at the site, and some pain or discomfort with the needle stick. There may also be some bruising at the site of the needle stick after the blood draw. If a central line (long-term IV line) is in place, we will draw blood samples from this line. Because the tumor tissue is collected during your child's regular clinically-indicated tumor surgery, there is no additional risk beyond that normally associated with the surgery. If the exome tests show a risk of developing a second cancer, a risk of cancer in family members, or a risk of developing other types of diseases unrelated to cancer, you might feel anxious or upset by the results. Your child's cancer doctor can discuss these risks with you and determine any medical follow-up that is indicated. There is also a potential risk in this type of genetic analysis for uncovering and conveying unwanted information regarding the biological relationship of parents and their children.

There is also the risk of a loss of privacy of your child's genetic information. The exome report will be placed in the electronic medical record and may be seen by your child's other doctors and health care workers. Health insurance companies may also have access to this information. There are laws to protect against the use of this information in making decisions about health insurance and employment. However, you may be asked to provide medical record information when your child applies for life insurance or disablity insurance.

As described earlier, all research genetic and clinical information for the study will be stored in a confidential access-controlled computer database. In addition, there are additional risks of loss of privacy if you consent to the sharing of this data with other BCM investigators, or to the de-identified release of your child's genetic information into scientific databases for other scientists to use. While we believe that the risks to your child and your family from participating in this study are low, we are unable to tell you exactly what all of the risks are. We believe that the benefits of learning more about cancer outweigh these potential risks.

Study staff will update you in a timely way on any new information that may affect your decision to stay in the study.

Potential Benefits
The benefits of participating in this study may be: Tumor and/or inherited mutations may be discovered by exome sequencing that would not have been found by other standard tests. Although we do not think that the mutations that are found are likely to change the planned cancer treatment for most children, it is possible that they may have implications for the clinical care of your child as well as other family members. For example, tumor mutations may rarely be identified that change the type of tumor that is being diagnosed. Alternatively, tumor mutations may be found that indicate that your child's

tumor may respond better or worse to a particular cancer treatment. We think this will be very rare for most children. It might be more common in cases where a child's tumor has returned after standard treatment and additional treatment options are being considered. In both cases, your child's cancer doctor may make changes to their treatment plan based on the results of tumor exome sequencing. Similarly, inherited mutations may be identified that indicate an increased risk of your child and potentially other family members to develop additional cancers and/or diseases other than cancer. If inherited mutations of this type are found, additional follow-up testing or treatment would be recommended if these interventions would be considered standard medical care. However, you may receive no benefit from participating.

Alternatives

The following alternative procedures or treatments are available if you choose not to participate in this study: You can have exome testing done outside the study, although the test is very expensive and your physician would need to order the test and the hospital would need to determine insurance coverage. This decision will not affect the care that your child receives for their cancer.

Subject Costs and Payments

You will not be asked to pay any costs related to this research. You will be given a $25 Amazon gift card after each completed parent survey for a total of three $25 Amazon gift cards. If you only complete some of these surveys, you will only be given a gift card for those surveys you complete.

If you are selected to participate in the study interviews, you will be paid $25 after each completed interview for a total of $75 for completion of the three interviews. If you only complete some of these interviews you will be paid only for those interviews that you complete.

This institution does not plan to pay royalties to you if a commercial product is developed from blood or tissue obtained from you during this study.

Patient Assent

If your child is the one invited to take part in this study you are signing to give your permission. Each child may agree to take part in a study at his or her own level of understanding. When you sign this you also note that your child understands and agrees to take part in this study according to his or her understanding.

Please print your child's name here ___________________________

INCORPORATION OF GENOMIC SEQUENCING INTO PEDIATRIC CANCER CARE

Background

You are being invited to participate in a research study. Your participation in this study is entirely voluntary. Should you choose to withdraw from this study, your decision will in no way affect the care that your child receives. Please read the consent form carefully and feel free to ask any questions before you agree to take part in the study. You will be given a copy of the consent form to keep if you decide to participate in the study.

Cancer is a disease caused by changes (mutations) in the genetic code of a cell. The genetic code is like a set of instructions that tell our cells how to grow properly. These instructions are referred to as genes. The mutations in genes found in cancer cells cause cells to grow and spread abnormally. Sometimes one of these mutations is present in every cell of the body ("inherited mutations") and can be found in a blood sample, while others are only found in the cancer cells ("tumor mutations"). A clinical test is now available to look for certain kinds of mutations in all 20,000 genes in a cell. This test is called "exome sequencing." It is important to understand that exome sequencing cannot find all types of mutations that might occur in your child's tumor or blood.

In this study we will use exome sequencing to look for inherited and tumor mutations occurring in your child. The exome sequencing test is not experimental and is performed in a clinically certified laboratory. However, most children with cancer do not have it done as part of their regular cancer care, and no one knows the best way to use the tests for cancer patients yet.

Purpose

The main goal of this study is to learn how to best report and use the clinical exome sequencing test results for childhood cancer patients at the Texas Children's Cancer Center (TCCC). We plan to study how these results can best be explained to physicians and parents of cancer patients. We want to learn what both groups prefer in receiving the results. We want to learn whether the results can help us to: (1) better understand the future cancer risk of childhood cancer patients and their family members so that better methods of screening and prevention can be developed and (2) make treatment decisions for our patients if their cancer returns.

A second goal of this study is to learn more about the biology of childhood cancer and develop better ways to prevent, detect, and treat these cancers. To complete this goal we will use additional research methods to find new inherited mutations and tumor mutations occurring in childhood cancers and we will combine these results with clinical information from patients' medical records. Since this is not the main goal of the study, you will be asked whether you want to participate in these additional studies.

Procedures

COLLECTION OF STUDY SAMPLES

As discussed during the consent conference for your child we asked for permission to use tumor and blood samples from your child for clinical exome sequencing. These will be obtained at (or near) the time of your child's tumor surgery and sent to the Baylor College of Medicine Whole Genome Laboratory (WGL) for exome sequencing. In addition, we are requesting blood samples from both parents (if available) so that the WGL can confirm inherited mutations identified in your child. The WGL handles your samples the way any regular medical samples are handled, following all standard procedures for sample tracking, storage, and confidentiality.

1. Parent blood sample(s). These samples are routinely requested by the WGL as part of your child's clinical exome sequencing test in order to confirm the inherited mutations found in your child. The parent blood samples are not required for enrollment on the study and do not undergo exome sequencing as part of your child's clinical test. Both parents (if available) will be asked to donate 2 teaspoons of blood. This blood draw will be performed either in the TCCC clinic or at the General Clinical Research Center within one month of study enrollment at a time arranged with you.

WILL I GET TO SEE THE RESULTS OF GENETIC TESTING?

Clinical Exome Test Results:

The exome test takes a long time to finish. We think it will be about 3 months after we take the sample until the results are ready. The results of the exome test will be given to your child's cancer doctor and also placed into your child's electronic medical record. Before your child's doctor meets with you to review the results, he or she will be able to discuss them with the investigators on this study who are experts in exome tests. Your child's cancer doctor will then explain the results to you and work with you to decide whether anything about your child's care should change based on the results. A genetic counselor will work with your cancer doctor to explain the inherited mutations to you. These results will be explained to you in one or two meetings scheduled at the same time as your child's regular clinic visits whenever possible.

These tests are very new and we do not know yet how to use exome testing to guide children's cancer treatment. Because the test takes so long to do, the results will not be available until after your child begins his or her treatment. We do not expect the test results to change anything your doctor planned to do for treatment. Therefore we do not think that getting the results is likely to make your child's treatment better or make it more likely for him or her to be cured. It is possible, however, that the results might reveal tumor or inherited mutations that matter to the clinical care of your child or family such as:

1. Tumor mutations that are normally found in a different type of tumor from what your child was diagnosed with. We think this will be rare, but if it does happen, your doctor may talk to you about changing your child's treatment plan.

2. Tumor mutations that make your doctor think your child's tumor will respond better or worse to a particular cancer treatment. We think this will be very rare for most children. It might be more common in cases where a child's tumor has returned after standard treatment and additional treatment options are being considered.

3. Inherited mutations that might affect how your child's body responds to certain medicines. This information might help your child's doctors choose the dose of some of your child's medicines.

4. Inherited mutations that cause your child to have an increased risk of developing other cancers (or a second cancer of the same type). This information may also provide information about the risk of cancer in close family members. For example, children and adults who inherit mutations in the gene called p53 are at increased risk for multiple different types of cancer. If an inherited mutation in this gene is found in your child then additional cancer screening would be recommended and close family members should also be checked for the mutation so that they can be screened as well if necessary.

5. Inherited mutations that are unrelated to cancer but provide information about a different medical condition for which treatment is available and recommended as standard medical care. For example, we might find a child who inherited a mutation that puts him/her at high risk of life-threatening bleeding from a blood vessel (called an aneurysm). If that type of mutation were found we would recommend additional follow-up testing and/or treatment as part of standard medical care.

If we find any of these kinds of mutations, your child's cancer doctor and a genetic counselor will explain them to you and work with you to determine the most appropriate screening or treatment (if any) for your child and family members.

Exome sequencing can also reveal information about whether your child is a carrier of a genetic disorder such as Cystic Fibrosis. This information may not affect your child's health but it may be helpful to know later in life. If a child has one of these kinds of mutations, he or she probably inherited it from a parent. Therefore the parents might want to be screened to see if there is a risk of having a child who has a genetic disorder. However, some people do not want to know this kind of information. This type of genetic information will only be included in your child's exome sequencing report if that option was chosen in your child's consent form when your child was enrolled on the study.

PROCEDURES TO HELP US LEARN THE BEST WAY TO COMMUNICATE CLINICAL EXOME TEST RESULTS

Because we want to learn more about how doctors and patients discuss exome test results, we will include the following activities if you participate in the study:

1. Audiorecording of clinic visit(s) where you learn about the results of exome sequencing. By studying how doctors and parents talk about genetic test results this will help us learn how to improve communication and understanding about these results between cancer doctors and patient families.

2. Parent surveys. Parent surveys will be conducted at three different time points during the study: soon after initial study enrollment, after disclosure of the exome sequencing results, and one year later. Each family will tell us which parent will fill out the survey at all three of the time points. The surveys will ask questions about your family history of cancer, your knowledge of genetics, how you like to learn information from doctors and how you like to make decisions. The surveys will take approximately 30 minutes to complete at your convenience.

3. Parent interviews. Some families will be randomly selected for more in-depth interviews at three different time points during the study: soon after initial study enrollment, after disclosure of the exome sequencing results, and one year later. Each family will select one parent toparticipate in all three interviews. The interviews will ask more detailed questions about what you expect to learn in this study, what information you are most interested in learning and how you make decisions. Each interview will take place at TCH, last approximately 1 hour, and be audiorecorded. We will try to schedule these interviews at times that would be convenient to you.

4. Physician interviews. We will interview the cancer doctors to find out what they find most useful in the exome reports and what they think are the best ways to talk about this information with families. We will also collect information from your child's medical records, including their age, ethnic background, diagnosis, disease history, medical treatments, and response to treatments. We hope this will help us understand whether the exome results are related to how children with cancer do.

ADDITIONAL LABORATORY RESEARCH STUDIES

After the exome sequencing test has been performed on your child's tumor sample, there may be some sample left over. We asked for permission to use this tumor sample for some additional genetic research tests. We also requested an additional blood sample from your child that will be used for research if permission was granted. Similarly, we would like to do additional genetic research tests on your blood samples and attempt to grow blood cells in the laboratory to use for tests of gene function. These tests could include exome sequencing, which is otherwise not performed on the parent samples. This may also include using newer methods of sequencing, or sequencing parts of the DNA that doesn't contain genes or studying the RNA and proteins that are coded by your DNA as well as grow blood cells in the laboratory to test for gene function.

Any results of these research tests would be preliminary and would not be reported to you or placed in the medical record. If research from this project is presented at research conferences or published in professional journals, we will not use any information such as name, address, telephone number, or social security number, included in the presentations or publications. If we identify a genetic change in your sample that we think is clinically important, we will share those results with you and make follow-up plans. It is important to realize that these will be research results, and must be confirmed in a clinical laboratory in order to be used for clinical purposes.

Please initial below if you consent to the collection of your (parental) blood sample for additional genetic research tests:

Yes_____ No_____ I consent to a blood sample (2 teaspoons) for additional genetic research tests.

Who will have access to your additional study information?

As we explained the clinical exome report of your child will be handled like any other clinical test and placed in the medical record and explained to you by your physician. For the the laboratory research studies, questionnaires, audiotapes and survey information, these results will also be stored in a confidential computer database along with all data about your child's biological samples and parents' biological samples (if provided). These biological samples and medical information will be labeled with a code, meaning that names and other identifying information are removed and samples are given computer-assigned numbers that the research team can use to access them. Only the investigators and selected research staff will be able to match the code to a particular person. Only the investigators and selected research staff will be able to access the database.

In order to speed research, other researchers would like to be able to study your child's blood and tumor samples as well as your own blood sample and have access to your genetic information so that they can compare it to the genetic information of others from other research studies and use it to answer future research questions. This information is most valuable when it is linked to some information about your medical and family history (clinical information). If you agree, de-identified research blood samples, as well as de-identified parts of your genetic information, and in some instances, clinical information, can be shared with other researchers at Baylor College of Medicine (BCM) who are conducting approved research studies. In addition, your genetic and clinical information can be shared by releasing it into scientific databases including those maintained by BCM and some maintained by the National Institutes of Health. These databases are restricted and can only be accessed

by approved researchers. Sharing this information will help advance medicine and medical research by allowing other researchers to use this information to help solve questions of what causes cancers and other diseases.

There is a risk that others will be able to trace this information back to you, which may impact the ability of you or other family members to obtain life insurance, health insurance, or other products that may take into account the result of these genetic studies. Nobody will be able to know just from looking at a database that the information belongs to you. However, because your genetic information is unique, there is a small chance that someone could trace the information back to you or close biological relatives. The current risk of this happening is very small, but may grow in the future as new ways of tracing the information back to you or your close biological relatives are developed. Thus, the risk that your privacy would be breached may increase over time. Researchers who access your genetic and clinical information will have a professional obligation to protect your privacy and maintain your confidentiality.

The decision of whether or not to allow genetic and clinical information about you that will be gathered in this research into scientific databases is completely up to you. There will be no penalty to you if you decide not to allow this information to be used. You can still participate in the main part of the study without allowing your information to be released outside the study. We strongly encourage you to discuss the decision about de-identified data release and its associated risks for you and your biological relatives with your family before you decide whether to consent to this part of the study.

Your child's research blood, leftover tumor samples, and genetic and clinical information will only be released to other approved research studies at BCM and/or into scientific databases if that option was chosen in your child's consent form when your child was enrolled on the study. Please initial below whether you agree to the de-identified release of your research blood samples and genetic and clinical information for other approved research studies at BCM and/or into scientific databases:

Yes_____ No_____ I consent to release of my research blood samples and genetic and clinical information to other BCM researchers conducting approved research studies.

Yes_____ No_____ I consent to release of my genetic and clinical information into scientific databases.

Permission for future recontact by study investigators

We will follow each child in the study for 2 years to determine if their cancer doctor has found the exome information useful in their care. In the future, it may be helpful to our research to be able to recontact you to obtain additional clinical information or to ask your permission to collect another research sample. Please initial below if you we may recontact you after those 2 years are completed to discuss these research opportunities.

Yes_____ No_____ I consent to be contacted in the future for research study purposes.

Can I change my mind after I agree to let our samples be used?

You can withdraw from this study for any reason at any time. If you decide to withdraw from the study, your samples (if applicable) will be discarded.

If you decide to withdraw from this study after your child's genetic code has been analyzed, your genetic information will be discarded and will not be used in this study. However, if your child's tumor and inherited exome sequencing reports have already been submitted into the medical record, it will not be possible to remove these reports from the medical record.

You can see and get a copy of your research related health information. Your research doctor may be able to provide you with part of your information while the study is in progress and the rest of your information at the end of the study.

The only physical risk of this study is related to obtaining the blood sample. The risk of drawing blood includes a small risk of bleeding or infection at the site, and some pain or discomfort with the needle stick. There may also be some bruising at the site of the needle stick after the blood draw.

If the exome tests show a risk for your child of developing a second cancer, a risk of cancer in family members, or a risk of developing other types of diseases unrelated to cancer, you might feel anxious or upset by the results. Your child's cancer doctor can discuss these risks with you and recommend an appropriate physician to decide on medical follow-up. There is also a potential risk in this type of genetic analysis for uncovering and conveying unwanted information regarding the biological relationship of parents and their children.

There is also the risk of a loss of privacy of your information. The exome report will be placed in the electronic medical record and may be seen by your child's other doctors and health care workers. Health insurance companies may also have access to this information. There are laws to protect against the use of this information in making decisions about health insurance and employment. However, you may be asked to provide medical record information when you or your child applies for life insurance or disablity insurance.

As described earlier, all research genetic and clinical information for the study will be stored in a confidential access-controlled computer database. In addition, there are additional risks of loss of privacy if you consent to the sharing of this data with other BCM investigators, or to the de-identified release of your genetic information into scientific databases for other scientists to use. While we believe that the risks to you and your family from participating in this study are low, we are unable to tell you exactly what all of the risks are. We believe that the benefits of learning more about cancer outweigh these potential risks.

Study staff will update you in a timely way on any new information that may affect your decision to stay in the study.

Potential Benefits

The benefits of participating in this study may be: The benefits of participating in this study may be: Tumor and/or inherited mutations may be discovered by exome sequencing that would not have been found by other standard tests. Although we do not think that the mutations that are found are likely to change the planned cancer treatment for most children, it is possible that they may have implications for the clinical care of your child as well as other family members. For example, tumor mutations may rarely be identified that change the type of tumor that is being diagnosed.

Alternatively, tumor mutations may be found that indicate that your child's tumor may respond better or worse to a particular cancer treatment. We think this will be very rare for most children. It might be

more common in cases where a child's tumor has returned after standard treatment and additional treatment options are being considered. In both cases, your child's cancer doctor may make changes to their treatment plan based on the results of tumor exome sequencing. Similarly, inherited mutations may be identified that indicate an increased risk of your child and potentially other family members to develop additional cancers and/or diseases other than cancer. If inherited mutations of this type are found, additional follow-up testing or treatment would be recommended if these interventions would be considered standard medical care. . However, you may receive no benefit from participating.

Alternatives
The following alternative procedures or treatments are available if you choose not to participate in this study: You can have exome testing done outside the study; although the test is very expensive and your own physician would need to order the test and the hospital would need to determine insurance coverage. This decision will not affect the care that your child receives for their cancer.

Subject Costs and Payments
You will not be asked to pay any costs related to this research. You will be given a $25 Amazon gift card after each completed parent survey for a total of three $25 Amazon gift cards. If you only complete some of these surveys, you will only be given a gift card for those surveys you complete.

If you are selected to participate in the study interviews, you will be paid $25 after each completed interview for a total of $75 for completion of the three interviews. If you only complete some of these interviews you will be paid only for those interviews that you complete.

This institution does not plan to pay royalties to you if a commercial product is developed from blood or tissue obtained from you during this study.
